# Efficacy of a Novel Food Supplement (Ferfer®) Containing Microencapsulated Iron in Liposomal Form in Female Iron Deficiency Anemia

**DOI:** 10.7759/cureus.4603

**Published:** 2019-05-07

**Authors:** Uzma Hussain, Kokab Zia, Riffat Iqbal, Muhammad Saeed, Nadia Ashraf

**Affiliations:** 1 Obstetrics and Gynecology, Lady Willingdon Hospital, Lahore, PAK; 2 Obstetrics and Gynecology, Avicenna Medical College, Lahore, PAK; 3 Obstetrics and Gynecology, Jinnah Hospital, Lahore, PAK

**Keywords:** iron deficiency anemia, non-pregnant women, reproductive age, microencapsulated iron, liposomal iron, iron pyrophosphate, taste, palatability, serum hemoglobin

## Abstract

Introduction

Iron deficiency anemia (IDA) is a highly afflicting condition which affects young children of growing age and reproductive age women in countries of lower economies. Conventional oral iron salts have poor absorption and gastrointestinal side effects. Microencapsulated liposomal iron pyrophosphate is a novel compound with enhanced palatability, higher bioavailability, and consequently increased adherence among people with IDA. This study aims to assess the efficacy of microencapsulated iron pyrophosphate sachets in non-pregnant women with IDA.

Methods

It was a 12-week long, open label clinical trial conducted with 558 IDA women. Participants were advised one sachet of microencapsulated liposomal iron pyrophosphate (Ferfer®) twice daily. At baseline, and every four-week interval, serum hemoglobin levels and taste tolerability were assessed. Data was entered and analyzed using SPSS v. 24 (IBM Corp, Armonk, NY, USA).

Results

Four hundred and thirty-seven women completed the trial. The mean serum Hb level at baseline was 8.71 ± 2.24 which increased to 10.47 ± 1.69 by the end of 12 weeks (p < 0.001).

Conclusion

Treatment of IDA with microencapsulated liposomal iron pyrophosphate sachets significantly increases serum hemoglobin levels in non-pregnant women of reproductive age.

## Introduction

According to the global anemia prevalence report published by the World health Organization (WHO) in 2011, Pakistani women of reproductive age (15-49 years) have a mean serum hemoglobin (Hb) level of 11.7 g/dL [1]. According to 2016 reports, 52.1% of non-pregnant Pakistani women have iron deficiency anemia (IDA) [2].

The two major causes of IDA in non-pregnant women of reproductive age are irregular menstrual bleeding (9-14%) [3] and a gastrointestinal (GI) source of bleed such as mucosal erosions (6-30%) [4]. Iron deficiency anemia is morbid as well as mortal [5]. In order to prevent its complications, supplementation of iron becomes essential. In non-pregnant women, mild, moderate, and severe IDA is classified as serum Hb level 11.9-10.0 g/dL for mild, 9.9-7.0 g/dL for moderate, and less than 7.0 g/dL for severe IDA [6].

The most appropriate form of iron supplementation is oral, unless it cannot be tolerated or absorbed. Worsening GI symptoms and major bowel surgeries which reduce iron absorption may require parenteral replacement [7]. Oral elemental iron of 30-60 mg/day is required for treating IDA in adults. The total duration of treatment is three months. Adequate response is gauged by a rise in serum Hb levels of 1 g/dL within one month of compliant therapy [8].

Although all of the available forms of oral iron supplementation may replenish the therapeutically required dose of elemental iron, the chief differences are in adherence to therapy. Inadequate treatment adherence due to non-availability, fear of side effects, particularly GI symptoms - nausea, vomiting, constipation, metallic taste [9, 10] - has been reported in the literature. Conventional iron supplements include ferrous sulphate with or without mucoproteose, ferrous sulphate (glycine), iron protein succinylate, ferrous gluconate and ferrous fumarate. Most of these salts injure the mucosal lining of the GI tract and cause other side effects such as constipation [11]. Along with poor absorption of conventional iron, side effect profile hampers treatment compliance [12].

Micronization and microencapsulation of iron into liposomes, in sachet form, is the most advanced approach to combat both issues with iron supplementation - absorption and tolerance. Micronization is the phenomenon of reducing individual particle size, thereby, increasing solubility, and hence, bioavailability, due to increased surface area to drug ratio. Microencapsulation is when the micronized iron is encapsulated inside a lipid bilayer, similar to the biological lipid bilayer. The outer phospholipid bilayer protects the inner iron core from enzymatic degradation in the mouth or stomach, and also prevents iron oxidation and degradation. Liposomes of iron, which are nanosized, have the advantage of quicker and better absorption with minimum oxidative damage and lower incidence of side effects. The lipid bilayer of liposomal iron gives the molecule stability and ability to release the contents gradually. Gradual release helps in better absorption. This sophisticated technology of liposomal encapsulation also prevents iron to come in direct contact with intestinal mucosal lining, hence improved tolerance [12-14].

With a shift of paradigm from prescription and over-the-counter drugs to nutraceuticals, a need was established to extract real world data regarding the safety and efficacy of various nutraceuticals. Iron pills are also important nutritional supplement and nutraceutical. Therefore, this study aims to assess the efficacy of microencapsulated iron pyrophosphate sachets in non-pregnant otherwise healthy women with iron deficiency anemia. Efficacy was measured in terms of increase in serum hemoglobin levels during the treatment period. This study also evaluated taste tolerability and palatability with this newer form of iron supplementation.

## Materials and methods

It was a multicenter, open label clinical trial conducted after approval from ethics review committee. It was registered at www.clinicaltrials.gov (NCT03112187). Otherwise healthy, non-pregnant women, of age 15 till 49 years, diagnosed with iron deficiency anemia who had their hemoglobin <8 to >5 g/dl were included after attaining written informed consent. All participants were given the right to refuse to participate in the study. Participants could also withdraw from the study at any time after informing the prescribing doctor. Furthermore, women who had a history of allergic reaction to iron supplementation, iron intolerance, hypersensitivity to vitamin C and vitamin B12, and individuals with causes of anemia other than iron deficiency were not included in the study.

A brief sociodemographic profile of all participants was recorded. It included their age, co-morbidities, and whether or not they have taken any iron supplements before. A brief gynecological assessment including menstrual frequency, painful menstruation and any history of anemia was also recorded.

The food supplement under consideration in this trial is microencapsulated iron pyrophosphate in liposomal form (Ferfer®: manufactured by PharmEvo Pvt. Ltd, Karachi, Pakistan). It is a water dispersible micronized source of iron that has been microencapsulated to enhance iron absorption and to lessen both GI aspect results and unwanted organoleptic attributes. Ferfer® is a 1.5-gm sachet which contains 14 mg iron in liposomal form, 80 mg vitamin C, and 2.5 mcg vitamin B12 with orodispersible granulate which immediately dissolves in the mouth without the want for water and therefore appropriate additionally for those who've problem swallowing.

Each patient completed four-doctor visits during the study period. On the first visit, women with serum Hb <8 to >5 g/dl were inducted and prescribed Ferfer®, twice daily for 12 weeks. Patients who were anemic from other causes were excluded by detailed history and relevant laboratory tests like B12 or folate deficiency. Complete blood count (CBC) with peripheral films and in some cases serum ferritin was used to diagnose patients. All subsequent visits were four weeks apart. On each of the four visits, serum hemoglobin level was measured. On second, third, and fourth visit, women were asked to document adverse effects and taste tolerability of Ferfer®. Taste tolerability was scored at a scale of 1-5, with 1 being the least tolerable and 5 being the most tolerable. Side effects including nausea, vomiting, bloating, abdominal cramps, early satiety, acid eructation/heartburn, sickness, loss of appetite, retrosternal discomfort, epigastric or upper abdominal pain, constipation were to be reported. Participants could also report any side effect other than these. The participants were also provided with a contact number to communicate in case any adverse effects occurred in between the monthly scheduled visits.

With an estimated anemia prevalence of 50%, the sample size calculated was 384 with 95% significance level. After consideration of dropout throughout the study period (12 weeks), 600 patients were enrolled in the trial. All data, including sociodemographic profile, weekly lab investigations, and adverse effects were entered and analyzed using SPSS version 24 (IBM Corp, Armonk, NY, USA). Frequency and percentages were computed for categorical variables including sociodemographic profile. Mean ± Standard Deviation (SD) was computed for numerical variables such as age, lab results, and tolerability symptoms. Paired T-test was used to compare means of haemoglobin at base line and after 12 weeks of therapy. P value of less than 0.05 was considered statistical significance. For the purpose of analysis, “all-treated population” was considered which included any subject who received at least one day therapy with agent.

## Results

At the start of the trial, there were 558 women who agreed to participate and fulfilled the inclusion criteria. Their sociodemographic and clinical profile is shown in Table 1.

**Table 1:** Baseline demographics and clinical characteristics of patients (n = 558).

Sociodemographic and clinical characteristics	Frequency (%) (N = 558)
Age in years (Mean ± SD)	33.2 ± 8.83
History of iron supplementation	92 (16.4)
Comorbidities	
Diabetes mellitus	16 (2.9)
Hypertension	53 (9.4)
Chronic heart disease	27 (4.8)
Angina	34 (6.1)
Previous history of iron deficiency anemia	180 (32.1)
Regular monthly menstruation for 5-7 days	212 (37.8)
Painful menstruation	212 (37.8)

By the end of the first month, 15 women were lost to follow up. By the end of the second month, 105 more women dropped out. With one more woman dropping out by the end of the trial period, there were 437 women who completed the entire trial. The mean serum hemoglobin level increased from 8.71 ± 2.24 g/dL at the start of the study to 10.47 ± 1.69 g/dL at the end of the trial. The mean taste tolerability also improved throughout the study period. The mean change in hemoglobin levels at each visit and the taste tolerability status is shown in Table 2.

**Table 2:** Change in hemoglobin level after therapy with microencapsulated iron in female iron deficiency anemia.

Scheduled visit	Number of subjects (N)	Hemoglobin (g/dL) (Mean ± SD)	Taste tolerability (Mean ± SD)
Start of therapy	558	8.71 ± 2.24	3.93 ± 0.93
Week 4	543	9.49 ± 1.81	3.94 ± 0.89
Week 8	438	9.80 ± 1.70	4.03 ± 0.82
Week 12	437	10.47 ± 1.69	4.05 ± 0.88

The mean change in serum hemoglobin level from the start of therapy till 12 weeks was statistically significant as shown in Table 3.

**Table 3:** Change in hemoglobin level at week 12 after therapy with microencapsulated iron in female iron deficiency anemia (n = 437).

Hemoglobin level	Mean ± SD (g/dL)	t value	95% CI	P value*
Start of therapy (n = 558)	8.71 ± 2.24	-25.6	-2.02 – (-1.74)	<0.001
At Week 12 (n = 437)	10.47 ± 1.69			

## Discussion

There is a significant increase in mean hemoglobin levels after 12-week supplementation with microencapsulated iron pyrophosphate sachets. Overall taste acceptability and palatability for this novel compound has already been studied [14].

Over the years, it has been established that the key component of successful iron replenishment is treatment adherence and compliance. Oral iron salts are absorbed via divalent metal transporter 1 (DMT-1). Conventional forms of oral iron salts have this advantage of cost effectiveness and extensive availability, nonetheless, the constraint of GI intolerance, especially metallic after taste, stands still [10, 12]. In an Ugandian study, only 12% pregnant women were compliant to their iron supplementation [9]. For Pakistan, reports indicate that only 38% women took iron and folic acid during their pregnancy [15].

Not many studies have been conducted to evaluate iron supplementation in non-pregnant women of reproductive age. In a 16-week long randomized double-blind placebo-controlled trial, non-pregnant iron deficient women were randomized to fruit juice fortified with placebo or microencapsulated iron pyrophosphate (18 mg/day elemental iron). There was significant improvement in total erythrocyte count, hematocrit count, red cell distribution width, serum ferritin and soluble transferrin receptor [16].

In another interesting study with post-menopausal iron deficient women, eight weeks of microencapsulated iron pyrophosphate (liposomal) was supplemented. There was significant rise in mean serum Hb, and hematocrit. They also reported higher tolerability with improved adverse effects as compared to previous conventional regimes of iron supplements taken by these women [17]. In a study with chronic kidney disease patients, parenteral iron supplementation was compared with oral liposomal iron and it was seen that with eight weeks of therapy, liposomal iron group had significant increase in serum Hb from baseline while the other group did not have significant rise in serum Hb [18]. Similarly, in this study, there was a significant increase in serum Hb levels with liposomal iron supplementation.

In another study conducted with microencapsulated iron pyrophosphate in liposomal form (Ferfer®), the mean taste score on Visual Analogue Scale (VAS) was 2.92 ± 2.44 with other forms of iron supplementation. VAS score increased to 7.66 ± 1.32 immediately after taking Ferfer® and to 7.96 ± 1.37 after five minutes [14].

Microencapsulated iron pyrophosphate in liposomal form is a novel advancement in management of iron deficiency anemia. This salt is “generally recognized as safe (GRAS)” by United States Food and Drugs Administration (USFDA) Code of Federal Regulation. Furthermore, European Food Safety Authority (EFSA) has also declared iron pyrophosphate to be a safe food additive [12]. Comparatively to conventional oral iron salts, microencapsulated liposomal iron has the highest bioavailability. It leads to quicker increase in serum hemoglobin levels, its taste has better palatability, and it doesn’t have unwanted effects such as heartburn, GI upset, and constipation.

Microencapsulated liposomal iron is an effective and efficacious means of iron replenishment in deficient populations. Further studies are recommended to evaluate the safety profile and adherence to therapy with microencapsulated liposomal iron as compared to other conventional oral iron salts. Although results of the current study are very favorable, however, there is a need for comparative studies with other forms of iron available. There is a need for much larger studies to generalize the findings.

## Conclusions

Iron deficiency anemia is an easily manageable yet highly prevalent condition. The population at risk includes children, adolescents, and women of reproductive age in underdeveloped and low economy countries. The key factor to efficacious treatment of IDA is adherence to therapy. Adherence is governed by higher palatability and lesser side effects. Microencapsulated liposomal iron pyrophosphate sachets come with enhanced palatability, higher bioavailability, and consequently increased adherence among people with IDA.

## References

[1] (2019). The global prevalence of anaemia in 2011. http://apps.who.int/iris/bitstream/handle/10665/177094/9789241564960_eng.pdfjsessionid=89EB1CFE1FAFDF2BFC118B431D3CE08C?sequence=1.

[2] (2019). Pakistan - Prevalence of anemia. https://www.indexmundi.com/facts/pakistan/prevalence-of-anemia.

[3] Fraser IS, Langham S, Uhl-Hochgraeber K (2009). Health-related quality of life and economic burden of abnormal uterine bleeding. Expert Rev Obstet Gynecol.

[4] Short MW, Domagalski JE (2013). Iron deficiency anemia: evaluation and management. Am Fam Physician.

[5] Khaskheli MN, Baloch S, Baloch AS, Baloch S, Khaskheli FK (2016). Iron deficiency anaemia is still a major killer of pregnant women. Pak J Med Sci.

[6] Kariyeva G, Magtymova A, Sharman A (2019). Anemia. The DHS Program, USAID.

[7] (2019). Nutritional anaemias: tools for effective prevention and control. http://apps.who.int/iris/bitstream/handle/10665/259425/9789241513067-eng.pdf.

[8] Baker RD, Greer FR (2010). Diagnosis and prevention of iron deficiency and iron-deficiency anemia in infants and young children (0-3 years of age). Pediatrics.

[9] Kiwanuka TS, Ononge S, Kiondo P, Namusoke F (2017). Adherence to iron supplements among women receiving antenatal care at Mulago National Referral Hospital, Uganda-cross-sectional study. BMC Res Notes.

[10] Gereklioglu C, Asma S, Korur A, Erdogan F, Kut A (2016). Medication adherence to oral iron therapy in patients with iron deficiency anemia. Pak J Med Sci.

[11] Yu PP, Chang YZ, Yu P (2015). Iron liposome: a more effective iron supplement for sports anemia and anemia of inflammation. J Pharma Care Health Sys.

[12] Biniwale P, Pal B, Sundari T (2018). Liposomal iron for iron deficiency anemia in women of reproductive age: review of current evidence. Open J Obstet Gynecol.

[13] Pisani A, Riccio E, Sabbatini M, Andreucci M, Del Rio A, Visciano B (2015). Effect of oral liposomal iron versus intravenous iron for treatment of iron deficiency anaemia in CKD patients: a randomized trial. Nephrol Dial Transplant.

[14] Siddiqui AS, Ashraf T, Imran U (2018). Palatability of micro-encapsulated iron pyrophosphate (Ferfer®). Int J Clinical Trials.

[15] Nisar YB, Dibley MJ, Mir AM (2014). Factors associated with non-use of antenatal iron and folic acid supplements among Pakistani women: a cross sectional household survey. BMC Pregnancy Childbirth.

[16] Blanco-Rojo R, Pérez-Granados AM, Toxqui L, González-Vizcayno C, Delgado MA, Vaquero MP (2011). Efficacy of a microencapsulated iron pyrophosphate-fortified fruit juice: a randomised, double-blind, placebo-controlled study in Spanish iron-deficient women. Br J Nutr.

[17] Pleşea-Condratovici A, Pleşea-Condratovici C, Rosoga N, Nedelcu N (2015). Efficacy and tolerability of a novel food supplement (Turbofer®) containing microencapsulated iron in liposomal form, in female iron deficiency anaemia. Prog Nutr.

[18] Visciano B, Nazzaro P, Tarantino G (2013). Liposomial iron: a new proposal for the treatment of anaemia in chronic kidney disease. (Article in Italian). G Ital Nefrol.

